
JOURNAL INFORMATION
==============================
NLM Title Abbreviation: ScientificWorldJournal
Journal ID: ScientificWorldJournal
NLM Journal ID: 101131163
Journal ID: TSWJ

The Scientific World Journal

ISSN: 2356-6140
EISSN: 1537-744X
Publisher: Wiley

ARTICLE INFORMATION
==============================
PMCID: PMC6083973
PMID: 30147511
DOI: 10.1100/tsw.2001.184
Article ID: 236438
Article version: 1
Subjects: Short Report

HIV Central Nervous System Infection in the Elderly

Denaro F.J. *
Bryant J.
Institute of Human Virology, University of Maryland, 725 West Lombard St. MD 21201, USA
*F.J. Denaro: denaro@umbi.umd.edu
Publication date: 2001
Electronic publication date: 2001 Jul 17
Volume: 1
First page: 51
Last page: 51
Copyright: Copyright © 2001 F.J. Denaro and J. Bryant.
Copyright year: 2001
License: This is an open access article distributed under the Creative Commons Attribution License, which permits unrestricted use, distribution, and reproduction in any medium, provided the original work is properly cited.
License URL: https://creativecommons.org/licenses/by/3.0/

Funding: National Institutes of Health NS31857 National Institutes of Health MH29494

==============================
Miami *Nature Biotechnology* Short Reports
*TheScientificWorld* (2001) 1 (S3), 51SR
ISSN 1532-2246; DOI 10.1100/tsw.2001.184

### HIV CENTRAL NERVOUS SYSTEM INFECTION IN THE ELDERLY

F. J. Denaro* and J. Bryant

Institute of Human Virology, University of Maryland, 725 West Lombard St. MD 21201
* Denaro@umbi.umd.edu

**INTRODUCTION.** Central nervous system pathology is a recognized consequence of HIV infection. This neuropathology results in HIV-1 Associated Dementia (HAD). Reported estimates for the frequency of HAD are as high as 31%. When the frequency of HAD is correlated with age, it is found that adults (Age: 60 years and greater) present with over twice the frequency of HAD as compared to young adults (ages 20-30) (1). There has been very little neuropathologic analysis of HIV/CNS infection in senior citizens. This may be because of the low number of HIV cases in this population at the present time. However, improved therapy is resulting in patients with increased age. Significantly, current studies have shown that while HAART can control many of the symptoms of AIDS, there is still a question of its effectiveness on preventing HAD (2). These points underscore the need for study of HIV/CNS infection in the aging and long-term survivors. It also raises the question as to why this particular population has an increased risk for developing HAD. There are several hypotheses that come to mind. One is the possibility of the combined effects of aging and HIV. Other mechanisms may also be operational. By studying the postmortem brain tissue from patients with AIDS (age greater than 60) it was hoped that further insight could be gained as to why the elderly present with increased risk for HAD.

**METHODS.** Tissue samples were obtained from the Institute for Brain Aging and Dementia at UC Irvine. These patients were diagnosed as HIV positive prior to autopsy. A clinical diagnosis of dementia was typical and a subsequent diagnosis of Alzheimer’s disease (AD) was often made. The average age was 70 years with a range of 58 to 83. There were five men and five women. Age matched controls were also studied. The frontal cortex, parietal cortex, caudate, putamen, and hippocampus were examined. Stains used were H&E, Nissl, Bielschowsky, PAS, and Immunocytochemistry to Beta amyloid, HIV and Nitrotyrosine (NT).

**RESULTS.** The neuropathology was interesting because of its marked severity and uniqueness. Figs. 1 and 3, display typical findings across brains: extremely extensive and floral, primitive plaques. Fig. 2, A plaque with HIV infected cells (possibly microglia) Beta Amyloid = brown stain, HIV=Black stain. Fig. 4, H&E, Neuron with a bilobular nucleus. Fig. 5, Nissl stain demonstrating atypical density of microglia in plaque. Fig. 6, Inflammatory changes. Fig. 7, Calcification found in the Caudate. Fig. 8, H&E Cortex, Spongiosis, Fig. 9, H&E Hippocampus, neuronal death. Fig 10, H&E of Plaque with lymphocyte cuffing, reactive astrocytes, Rosenthal fibers, swollen endothelia cells, microglia and neuron. Fig. 11, Immunoreactivity for NT. NT is marker for free radical damage.

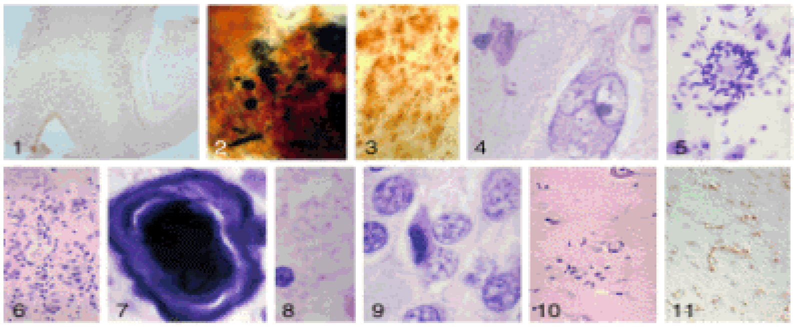

**DISCUSSION.** The neuropathology found was consistent with HIV/CNS infection and AD. What is of interest is when these two pathologies combine or overlap. For example, HIV infected cells were found in the AD plaques, and extensive plaque formation was found in areas of HIV associated inflammatory changes. In both areas NT immunoreactive was evident. The complementary nature of these two-diseases might reinforce the likelihood of neuronal death. The clustered microglia typically found in AD may eventual become infected with HIV. This in turn could facilitate Nitric oxide production and result in reactive radical damage.

**ACKNOWLEDGEMENTS.** NIH grants NS31857 and MH29494.

**REFERENCES.**

1. Janssen, R.S. et al. (1992) Neurology 42, 1472-1476
2. Enzensberger, W. (1999) Eur. J. Med. Res. 11, 456-462
